# Comparative transcriptome analysis reveals carbohydrate and lipid metabolism blocks in *Brassica napus* L. male sterility induced by the chemical hybridization agent monosulfuron ester sodium

**DOI:** 10.1186/s12864-015-1388-5

**Published:** 2015-03-17

**Authors:** Zhanjie Li, Yufeng Cheng, Jianmin Cui, Peipei Zhang, Huixian Zhao, Shengwu Hu

**Affiliations:** State Key Laboratory of Crop Stress Biology in Arid Areas, Northwest A&F University, Yangling, Shaanxi 712100 P. R. China; College of Life Sciences, Northwest A&F University, Yangling, Shaanxi 712100 P.R. China; College of Agronomy, Northwest A&F University, Yangling, Shaanxi 712100 P.R. China

**Keywords:** *Brassica napus* L., Chemical hybridization agent, Male sterility, Monosulfuron ester sodium, Expression profile, Carbohydrate and lipid metabolism

## Abstract

**Background:**

Chemical hybridization agents (CHAs) are often used to induce male sterility for the production of hybrid seeds. We previously discovered that monosulfuron ester sodium (MES), an acetolactate synthase (ALS) inhibitor of the herbicide sulfonylurea family, can induce rapeseed (*Brassica napus* L.) male sterility at approximately 1% concentration required for its herbicidal activity. To find some clues to the mechanism of MES inducing male sterility, the ultrastructural cytology observations, comparative transcriptome analysis, and physiological analysis on carbohydrate content were carried out in leaves and anthers at different developmental stages between the MES-treated and mock-treated rapeseed plants.

**Results:**

Cytological analysis revealed that the plastid ultrastructure was abnormal in pollen mother cells and tapetal cells in male sterility anthers induced by MES treatment, with less material accumulation in it. However, starch granules were observed in chloroplastids of the epidermis cells in male sterility anthers. Comparative transcriptome analysis identified 1501 differentially expressed transcripts (DETs) in leaves and anthers at different developmental stages, most of these DETs being localized in plastid and mitochondrion. Transcripts involved in metabolism, especially in carbohydrate and lipid metabolism, and cellular transport were differentially expressed. Pathway visualization showed that the tightly regulated gene network for metabolism was reprogrammed to respond to MES treatment. The results of cytological observation and transcriptome analysis in the MES-treated rapeseed plants were mirrored by carbohydrate content analysis. MES treatment led to decrease in soluble sugars content in leaves and early stage buds, but increase in soluble sugars content and decrease in starch content in middle stage buds.

**Conclusions:**

Our integrative results suggested that carbohydrate and lipid metabolism were influenced by CHA-MES treatment during rapeseed anther development, which might responsible for low concentration MES specifically inducing male sterility. A simple action model of CHA-MES inducing male sterility in *B. napus* was proposed. These results will help us to understand the mechanism of MES inducing male sterility at low concentration, and might provide some potential targets for developing new male sterility inducing CHAs and for genetic manipulation in rapeseed breeding.

**Electronic supplementary material:**

The online version of this article (doi:10.1186/s12864-015-1388-5) contains supplementary material, which is available to authorized users.

## Background

In plants, the hybrid F1 progeny usually exhibits heterosis (hybrid vigour) relative to the inbred parents [1,2]. Accordingly, the productivity of many crops has been boosted by introducing hybrid varieties [3]. An effective pollination control system is a prerequisite for heterosis utilization. In 1950, it was reported that the plant growth regulator maleic hydrazide can induce male sterility in corn plants [4,5]. This initial finding led to the induction of male sterility by a chemical hybridization agent (CHA), which became an important tool for crop heterosis. CHAs are not restricted to particular species and do not require the laborious practice of transferring sterility and fertility genes from one species/line to another, unlike the other two popular pollination control systems in hybrid breeding, i.e. cytoplasmic male sterility (CMS) and nuclear male sterility (NMS). In addition, CHAs enable breeders to develop hybrids with a higher heterosis level in a shorter time [3]. The technique is now widely used in crops heterosis, particularly in rapeseed (*Brassica napus* L.) [6,7]. Till date, several dozens of commercial hybrids based on CHA-induced male sterility have been registered according to the data from the bulletins of Chinese National Crop Variety Approval Committee. The availability of safe and selective chemicals capable of inducing male sterility without causing any significant adverse effect on plant growth and development has been the necessary prerequisite in the pursuit of this approach. We previously found that monosulfuron ester sodium (MES) can induce complete male sterility in rapeseed at a concentration below 1% of that required for its herbicide activity and it has no significant influence on plant vegetative growth [8].

In the herbicide field, sulfonylurea is well known for its eco-friendly, extreme low toxicity towards mammals, and ultralow dosage application [9]. MES is a new sulfonylurea herbicide that inhibits acetolactate synthase (ALS, EC4.1.3.18, also known as acetohydroxyacid synthase, AHAS), an enzyme in the first step of the branched-chain amino acids (BCAAs; including valine, leucine, and isoleucine) biosynthesis pathway [9]. Plant ALSs are encoded by nuclear genes, and their N-terminal signal peptide sequence is required for translocating the protein to the chloroplast [10]. In addition, ALS is the target of four other classes of herbicides in addition to the sulfonylurea class, including triazolopyrimidines, pyrimidinylthiobenzoates, sulfonylamino-carbonyltriazolinones, and imidazolinones [11]. Several ALS inhibitor herbicides are exploited as CHAs in crop breeding [12]. Previous studies suggested several biochemical and physiological effects as consequence of the primary action of ALS inhibitors when it was used at lethal concentration: a quick accumulation of pyruvate (the main substrate of ALS) [13,14]; increase in free amino acid pool likely through protein turnover [15-18]; a rapid accumulation of carbohydrate in leaves [19] related to decreased photoassimilate translocation to sink tissues [20] due to a decreased sink strength [21]; and induction of fermentative metabolism [13,22]. Two other studies reported genome-wide gene expression responses to different ALS-inhibitor herbicides in *Arabidopsis thaliana* using the Affymetrix ATH1 microarray [23,24]. Till date, very few studies were carried out to investigate the mechanism of ALS inhibitor CHAs inducing male sterility [8].

In flowering plants, the development of the male gametophyte occurs in the anther, and it is a well-programmed and elaborate process [25-27]. In *Arabidopsis*, anther development consists of two phases divided into 14 stages [27,28]. During phase I, from stage 1 to 8, the four lobes of the anther are formed, each containing reproductive cells (microspore mother cells) and nonreproductive cell layers. The lobe is organised and includes the following from the exterior to the interior: the epidermis, endothecium, middle layer, and tapetum [27,28]. The developing pollen is immersed in locular fluid containing nutrients such as sugars and lipids from the sporophytic (somatic) tissue tapetum [29]. The early stages of pollen development are characterized by active growth and high metabolic activity in the anther. Thus, anthers have the highest sink strength in developing flowers, and large amounts of sugars are mobilized to anthers for supporting their early development [30]. During phase II, microspores undergo meiosis to form the tetrads enclosed in a thick shell composed of a callose (β-1,3 glucan) wall and a pollen mother cell (PMC) wall composed of cellulose, hemicelluloses, and pectins [31]. The timely degradation of the callose and PMC walls is critical for microspore release from the tetrads [31]. At least three cell wall enzymes are involved in this process, including β-1,3-glucanase [32,33], endocellulase [34,35], and polygalacturonase (PG) [36]. During the maturation of pollen grains, the grains accumulate an energy reserve in the form of starch for germination and starch thus serves as a marker of pollen maturity [37]. On the other hand, at the late unicellular stage or early bicellular stage, tapetal cells degrade and their remnants are deposited on the pollen exine [38]. Sporopollenin, the major component of exine, is a complex polymer primarily composed of fatty acids and phenolic compounds [39]. Therefore, the biosynthesis and export processes of fatty acids are essential for exine formation. The development of microgametogenesis involves numerous genes expression and a large part of the metabolism coordinated by a complex regulation network in both somatic and gametophytic cells [40].

To better understand the mechanism how ALS inhibitor CHAs induced male sterility in rapeseed, we treated the rapeseed plants at the bolting stage with 0.1 μg mL^−1^ MES to induce male sterility during the entire flowering period without significantly affecting other tissues growth and development. The objectives of this study were to investigate 1) whether the ultrastructure of the anthers was affected in MES treated plants 2) which set of genes differentially expressed might be associated with the ultrastructure changes of MES treated anthers 3) how these cytological and transcriptome changes relate to modification of physiological processes in rapeseed plants after MES treatment. This study will provide some clues to the mechanism of MES inducing male sterility, and provide some potential targets for developing new CHAs and for genetic manipulation during rapeseed breeding.

## Results

### Cytological studies reveal that MES treatment affects the plastid ultrastructure and metabolite accumulation in the developing anthers

**Figure 1:**
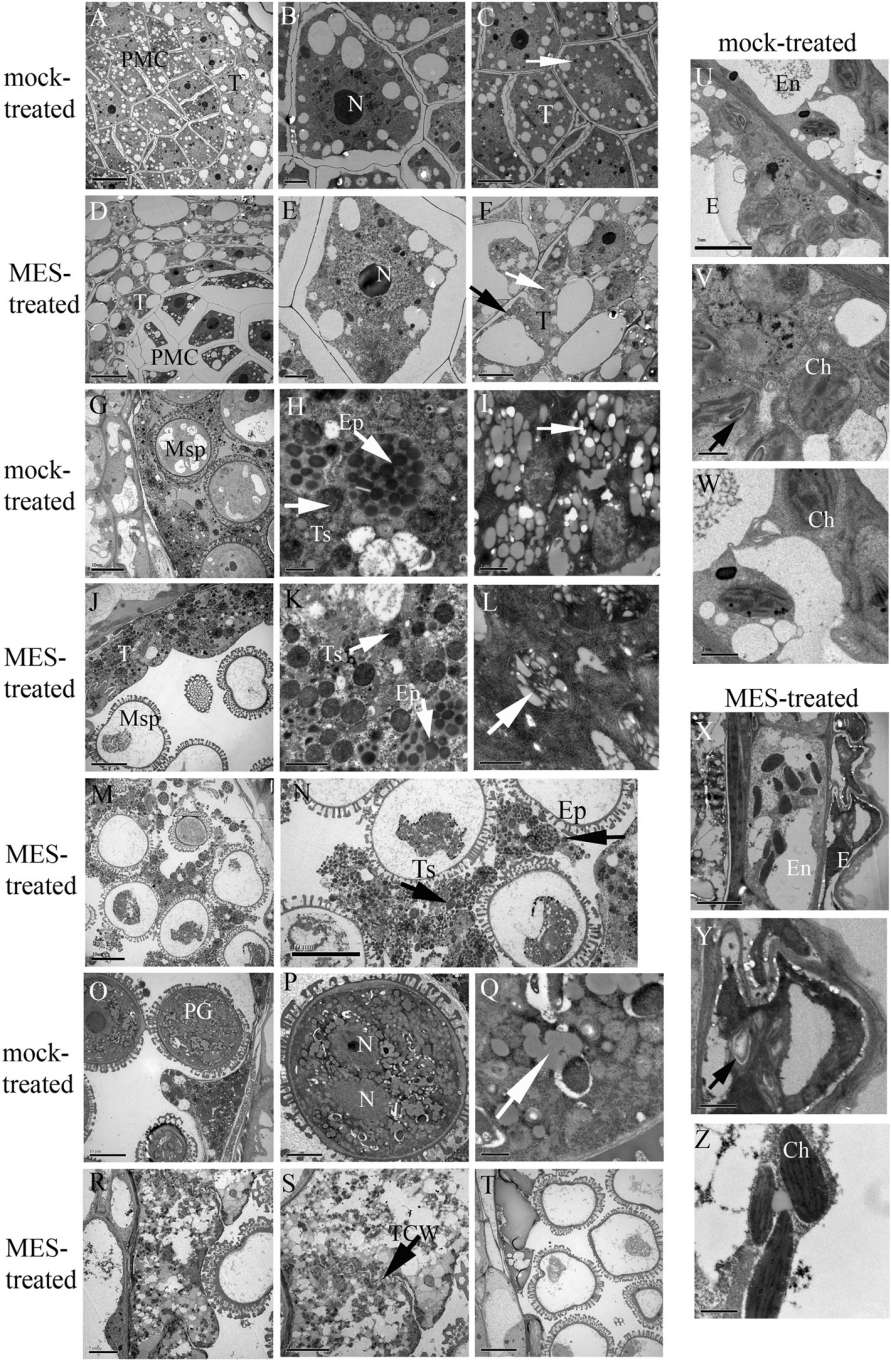
**Transmission Electron Microscope (TEM) micrographs of the anthers from the mock-treated (fertile) and MES-treated (sterile) plants. (A)** The fertile anthers at pollen mother cell (PMC) stage; **(B)** Enlarged fertile meiocytes in **(A)**; and **(C)** Enlarged fertile tapetum in **(A)** showing numerous plastids dispersed in cytoplasm (white arrow). **(D)** The sterile anthers at PMC stage; **(E)** Enlarged sterile meiocytes in **(D)** showing less plastids in condensed cytoplasm separated from the cell wall; **(F)** Enlarged sterile tapetum in **(D)** showing little abnormal plastids (white arrow) and more large vacuoles in cytoplasm, and with a little plasmolysis at meiocyte side (black arrow). **(G)** The fertile anthers at vacuolated-microspore stage; **(H)** The degraded tapetum in **(G)** showing elaioplasts and tapetsomes with abundant lipids; **(I)** Plastids in tapetum located in a crown showing filled with globular low electron-dense metabolites and surrounded by rich endoplasmic reticulum (ER). **(J)** The sterile anthers at vacuolated-microspore stage (type I); **(K)** The undegraded tapetum in **(J)** showing elaioplasts and tapetsomes with abundant lipids; **(L)** Plastids in tapetum located in a crown showing irregular shaped low electron-dense material. **(M)** The sterile anthers at vacuolated-microspore stage (type II); **(N)** The degraded tapetum in **(M)** showing scattered elaioplasts and tapetsomes with fuzzy structure; **(O)** The fertile anthers at mature pollen grain stage; **(P)** The pollen grain in **(O)** showing profuse globular particles; **(Q)** The enlarged globular particles in **(P)**. **(R)** The sterile anthers at mature pollen grain stage (type II); **(S)** The undegraded tapetum in **(R)** died but cell wall still existed (black arrow); **(T)** The sterile anthers at mature pollen grain stage (type I). **(U)** The epidermis and endothecium cells in fertile plants at vacuolated-microspore stage; **(V)** The epidermis cells in **(U)** showing normal oval-shaped chloroplastids with distinct thylakoid structure and little starch granules in thylakoid; **(W)** The endothecium cells in **(U)** showing oval-shaped chloroplastids with distinct thylakoid structure. **(X)** The epidermis and endothecium cells in sterile plants at vacuolated-microspore stage; **(Y)** The epidermis cells in **(X)** showing abnormal chloroplastids with large starch granules in thylakoid; **(Z)** The endothecium cells in **(X)** showing fusiform-shaped chloroplastids with linear thylakoid structure. PMC, pollen mother cell; N, nucleus; T, tapetum; Msp, microspore; Ep, elaioplast; Ts, tapetosome; PG, pollen grain; TCW, tapetum cell wall; E, epidermis; En, endothecium; Ch, chloroplast. Scale bars = 10 μm (A, D, G, J, M, N, O and T), 5 μm (C, F, P, R, S, U and X), 2 μm (B, E and K), and 1 μm (H, I, L, Q, V, W, Y and Z).

We previously showed that MES treatment causes two typical defects in sterile anthers: type I with early broken down tapetum at the PMC stage and type II with abnormal nondegraded tapetum at the mature pollen stage [8]. To better understand these phenomena, we observed the ultrastructure of fertile and sterile anthers from the mock-treated and MES-treated plants, respectively, during their development. The results showed that MES treatment affected the plastid ultrastructure and metabolite accumulation in the developing anthers (Figure 1). At the PMC stage, numerous plastids are dispersed in the cytoplasm of PMCs and tapetal cells in the mock-treated plants (Figure 1A–C). However, serious plasmolysis in PMCs and slight plasmolysis in tapetal cells were observed in the MES-treated male sterile plants (Figure 1D, E, and black arrow in 1F), and the cytoplasm of meiocytes and tapetal cells exhibited low electron density, with less plastids dispersed in them. At the vacuolated-microspore stage, the tapetum cells began to degrade and a number of elaioplasts and tapetosomes with abundant lipid compounds were formed in the tapetum of the mock-treated fertile anther (Figure 1G, H, white arrow). Besides, another type of plastids located in a crown, started to accumulate low electron-dense material and was surrounded by the rich ER (Figure 1I, white arrow). In contrast, in the MES-treated sterile plants, two types of abnormal tapetum were observed, as shown by Cheng *et al*. (2013) [8]. In type II abnormal tapetum, a number of elaioplasts and tapetosomes were formed, as seen in fertile plants; however, the tapetum cells did not degrade, the crowned plastids showed an irregular shape and were not well developed (Figure 1J, K, L). Type I abnormal tapetum was degraded and released noncompact elaioplasts and low electron-dense tapetosomes (Figure 1M, N). At the mature-pollen stage, the fertile pollen grains showed profuse globular particles (Figure 1O, P, Q); however, the sterile pollen grains were almost empty, type II tapetum still showed an intact and visible tapetal cell wall (Figure 1R, S), and type I tapetum showed solidified bulks (Figure 1T).

Furthermore, at the vacuolated-microspore stage, the chloroplastids in the epidermis and endothecium cells of the MES-treated plants exhibited defects. In fertile plants, the epidermis and endothecium cells showed normal oval-shaped chloroplastids with a distinct thylakoid structure and little starch granules in the thylakoid (Figure 1U, V, W); however, in the MES-treated sterile plants, the chloroplastids of the epidermis cells showed large starch granules in the thylakoid and the endothecium cells displayed fusiform-shaped chloroplastids with a linear thylakoid structure (Figure 1X, Y, Z).

### Identification of transcripts differentially expressed between the MES-treated and mock-treated rapeseed plants

**Figure 2:**
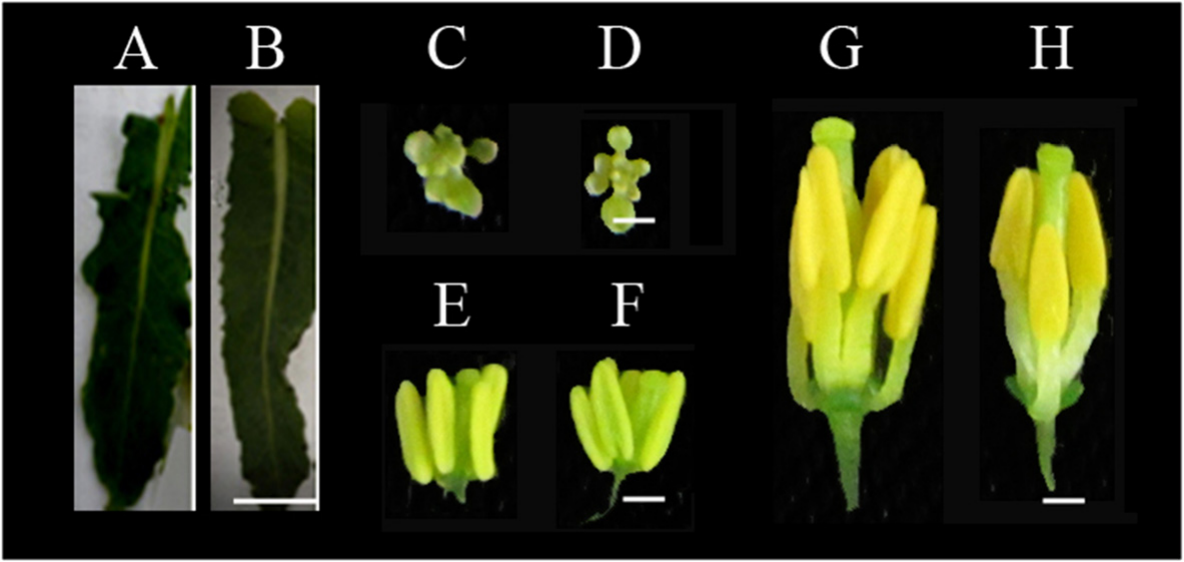
**Photographs of the leaves and developmental anthers for microarrays from the mock-treated (A, C, E, G) and MES-treated (B, D, F, H) plants.** **A-B**, young leaves from the main inflorescences (Ls); **C-D**, small buds (SBs); **E-F**, anthers from middle buds (An-MBs); **G-H**, anthers form large buds (An-LBs). Scale bar in leaves was 1 cm, scale bars in SBs, An-MBs, and An-LBs were 1 mm.

To obtain genome-wide gene expression profiles in the MES-treated and mock-treated plants of *B. napus*, the Agilent Single Channel *Brassica* Oligo Microarray (4 × 44 K) was used. Three independent biological replicates of four pairs of tissues (organs) from the MES-treated and mock-treated plants were collected for gene expression analysis, resulting in a dataset of 24 microarrays. The four tissues (organs) (Figure 2) included the leaves from main inflorescences (Ls), the small buds less than 1 mm in length containing microgametocytes before and during pollen mother stage (SBs), the anthers from middle buds with length between 1 mm and 3 mm containing microgametocytes from meiosis to early uninucleate microspore stage (An-MBs), and the anthers from large buds more than 3 mm in length containing microgametocytes from vacuolated stage to mature pollen stage (An-LBs). The data quality was assessed using two measurements: (1) correlation coefficients between biological replicates, which ranged from 0.8495 to 0.9906, with a mean of 0.9447 (Additional file 1), and (2) quantitative real time RT-PCR (qRT-PCR), which was performed on 62 randomly selected genes. The results of qRT-PCT analysis showed a high degree of concordance (R^2^ = 0.8775) with microarray results (Additional file 2). Taken together, these demonstrated that the microarray results obtained in this study were reliable.

**Figure 3:**
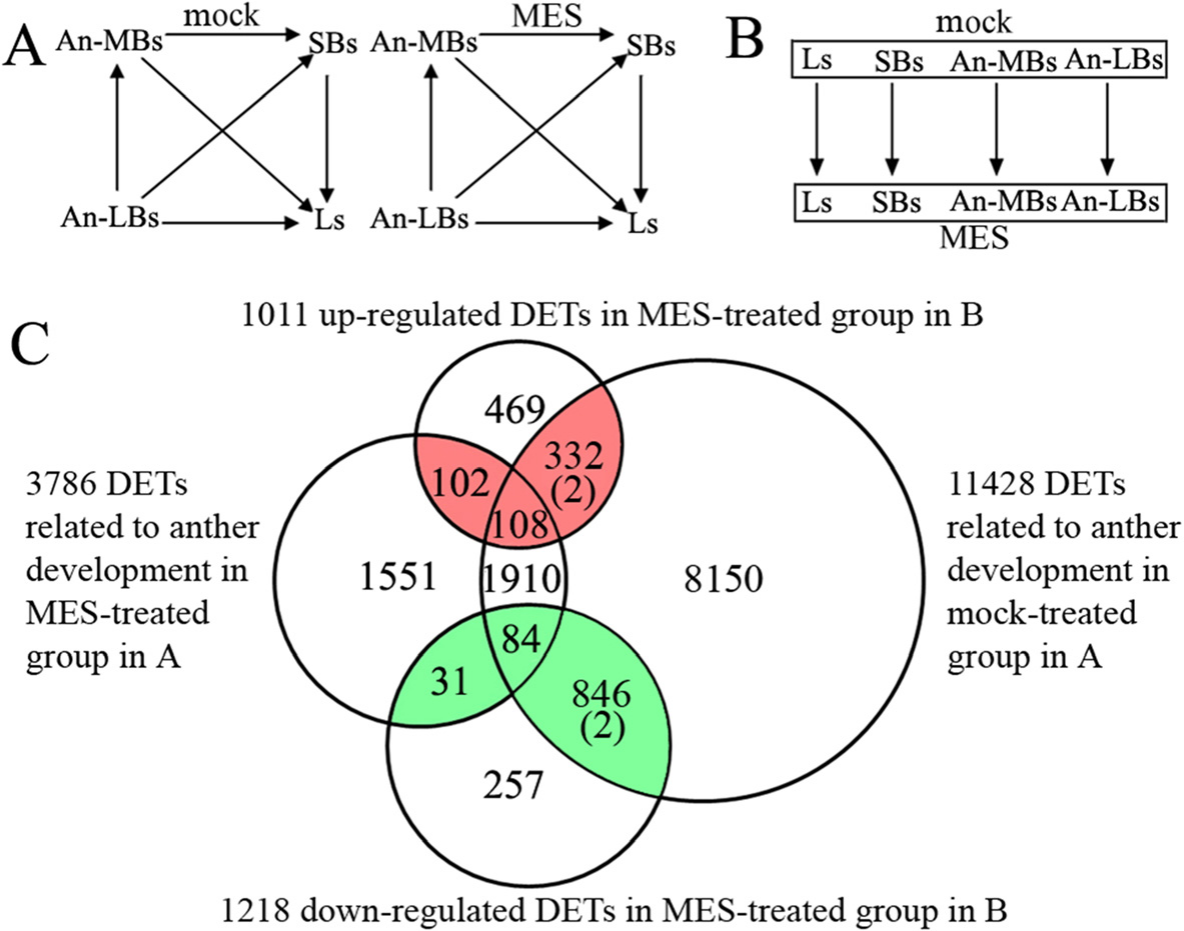
**Strategies for identification of differentially expressed transcripts (DETs) involved in microgametogenesis between the MES-treated and mock-treated plants by two sets of student’s t-test comparisons. (A)** Comparisons within groups. The pair-wise comparisons of Student’s t-test between tissues (organs) were carried out within mock-treatment groups and MES-treatment groups, respectively, to detected DETs related to anther development under mock-treatment (control, fertile) and MES-treatment (male sterile) conditions. The criteria for screening DETs were p-value <0.001 and fold change ≥ 2. mock, mock-treatment; MES, MES-treatment; **(B)** Comparisons between the MES-treated and mock-treated groups. The pair-wise comparisons of Student’s t-test were performed between the corresponding tissues (organs) of the mock-treated group and the MES-treated group to identify DETs related to MES-treatment. The screen criteria were same as above. **(C)** Venn diagram showing the DETs involved in microgametogenesis between the MES-treated and mock-treated groups. Comparisons within groups produced two sets of DETs, development-related genes in the MES-treated plants and in the mock-treated plants (the left and right cycles). Comparisons between groups also produced two sets of DETs, up-regulated genes and down-regulated genes in MES-treated group (the up and down cycles). These four sets of DETs were all collected, respectively. The common sections (totally 1501 unique DETs, the red and green parts, (2) indicates 2 DETs existing in the both data sets) were considered to be anther development-related genes affected by MES-treatment.

To identify differentially expressed transcripts (DETs) between the MES-treated and mock-treated groups involved in microgametogenesis, two sets of Student’s t-test comparisons were performed (Figure 3). Firstly, vertical comparisons (comparisons within groups, Figure 3A) were conducted to identify DETs involved in anther development, 11,428 and 3,786 DETs being identified within the mock-treated group and MES-treated group, respectively (right and left circle in Figure 3C). Secondly, horizontal comparisons (comparisons between groups, Figure 3B) were conducted to identify DETs caused by MES-treatment in the tissues (organs) tested, 1011 up-regulated and 1218 down-regulated transcripts being identified in the four-pair tissues (organs) (up and bottom circle in Figure 3C). The common DETs identified in both the vertical and horizontal comparisons described above were considered as anther development related genes affected by MES treatment (Figure 3C). Therefore, 102 + 108 + 332 and 31 + 84 + 846 DETs (red and green parts in venn diagram in Figure 3C) corresponding to 1501 unique DETs (Additional file 3) were selected for further analysis in this study, in order to reveal the alteration of gene expressions induced by MES treatment in the tissues (organs) tested. Distribution of these 1501 DETs indicated that over 64% (961/1501) were down-regulated and only 36% (542/1501) were up-regulated in at least one tissue (organ) in the MES-treated plants (Table 1). In addition, small fractions of these DETs were in Ls and SBs (77 and 60, respectively) with 2 ~ 5-fold change (up-regulation or down-regulation), while the majority were found in An-MBs and An-LBs (127 and 898, respectively) mainly with 10-fold or more down-regulation. This suggested that MES treatment led to expression alterations of a small number of genes in Ls and SBs of rapeseed plants and a large number of genes in An-MBs and An-LBs.

**Table 1:** **Distribution of differentially expressed transcripts (DETs) in MES-treated rapeseed plants**

**Fold change**	**Ls**	**SBs**	**An-MBs**	**An-LBs**	**Total unique**
**Up-regulated**					
**2 ~ 5**	24	31	39	280	
**5 ~ 10**	2	6	25	66	
**≥10**	0	2	21	69	
**Total-up**	26	39	85	415	542
**Down-regulated**					
**2 ~ 5**	48	18	14	136	
**5 ~ 10**	3	5	3	79	
**≥10**	0	5	59	608	
**Total-down**	51	28	76	823	961
**Total**	77	67	161	1238	1501*

*two DETs (A_46_P229574, A_46_P381563) existed both in the up-regulated and down-regulated probe sets.

### Subcellular localization and functional category analysis of differentially expressed genes

To reveal the functions of these DETs identified above, we annotated them by BLASTN against *Arabidopsis* Information Resource (TAIR, http://www.arabidopsis.org/Blast/index.jsp), considering the limited information of gene functional annotations in *B. napus* and the sufficient information in *Arabidopsis* as well as very high coding-sequence similarity (approximately 85%) between these two species [41]. Of the 1501 DETs, 1379 (91.87%) were highly similar to 1807 *A. thaliana* genes (AGI identifiers) (BLSATN; E-value < 10^−5^ for nucleic acids) and could be thus annotated. The remaining 122 DETs did not find a close orthologue in the TAIR database and therefore was not be annotated (Additional file 4).

**Figure 4:**
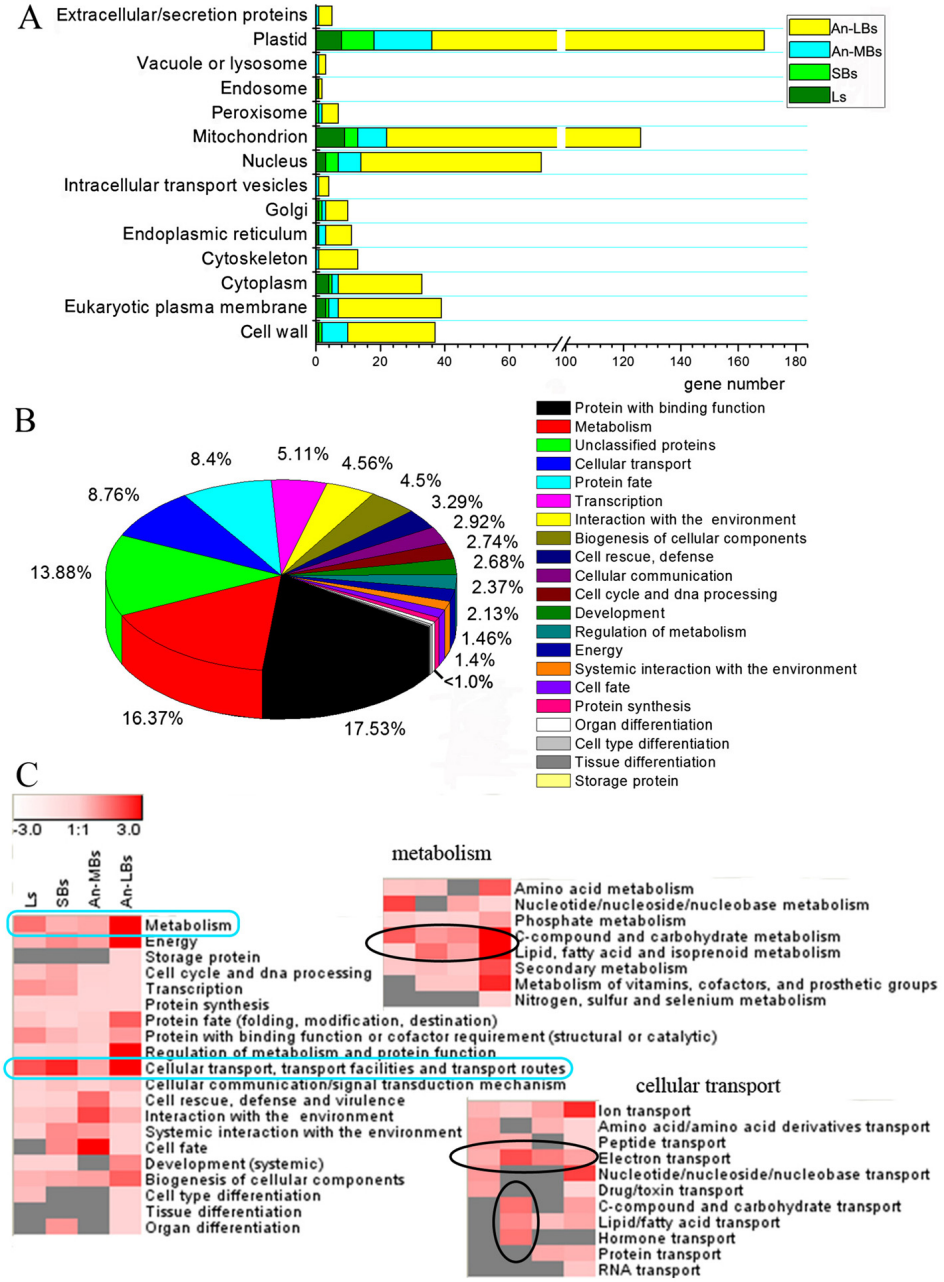
**Subcellular localization and functional categories of the 1087 differentially expressed unigenes between the MES-treated and the mock-treated rapeseed plants. (A)** Subcellular localization, **(B)** Functional categories, **(C)** Enrichment analysis of the functional categories list in B. The scale bar indicates -log (P-value), with highly enriched categories in red color, and invalid values in gray, whereas the P-value was calculated according to a hypothesis test using cumulative hypergeometric distribution. Left panel, enrichment analysis of all the unigenes in functional categories listed in B, except for those in unclassified category; the right panels, enrichment analysis of the sub-categories from metabolism and cellular transport (blue rectangles), respectively. The prominent enriched sub-categories were circled in black ellipses.

The localization of these DETs might provide clues to where they function. To gain insight into the biological functions of these 1087 annotated unigenes, subcellular localization and functional category analysis were conducted according to the information from Munich Information Center for Protein Sequences (MIPS) (Figure 4). The 1087 unigenes could be distributed into 14 subcellular localizations, with the largest three categories being the plastid/chloroplast (31.95%), mitochondrion (23.82%), and nucleus (13.23%), followed by the eukaryotic plasma membrane (7.37%), cell wall (6.99%), and cytoplasm (6.24%) (Figure 4A). Furthermore, the 1087 unigenes were classified into 21 functional categories, and the top five categories were proteins with binding functions, metabolism, unclassified proteins, cellular transport, and protein fate, representing approximately 70% of the total differentially expressed unigenes (Figure 4B). In order to understand the functions of DETs in different tissues, we performed the category enrichment analysis in four tissues separately (Figure 4C). The results of the functional category (Figure 4B) and enrichment analysis (Figure 4C) suggested that two categories, namely metabolism and cellular transport, were particularly affected by the treatment. They were not only overrepresented in all four tissues but also in the top four functional categories for relative abundance. The other overrepresented classes with only few genes falling in a certain functional category, including energy, cell rescue, and interaction with the environment etc., won’t be given further consideration in this study. Furthermore, detailed subcategory analysis of metabolism revealed that carbohydrate metabolism and lipid metabolism were significantly enriched (top of the right panel in Figure 4C), while detailed subcategory analysis of cellular transport exhibited that electron transport was enriched in all the tissues, along with carbohydrate transport, lipid transport, and hormone transport that were highly enriched in SBs (bottom of the right panel in Figure 4C).

**Figure 5:**
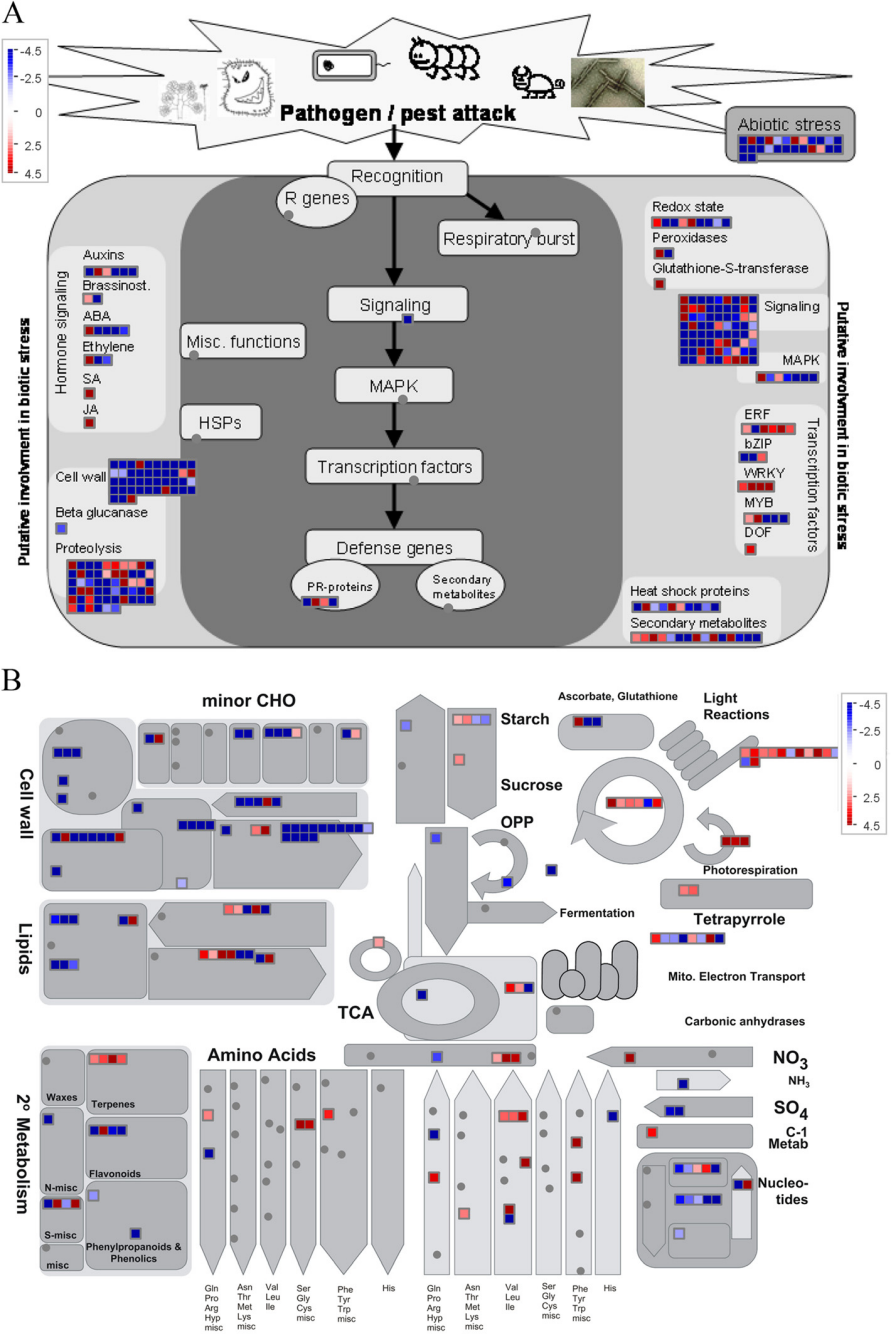
**Transcripts involved in stress (A) and metabolism (B) assigned by MapMan in rapeseed leaves and developmental anthers treated by MES.** Positive fold change values (red) indicate up-regulation, whereas negative fold change values (blue) denote down-regulation. Color saturates at 4.5-fold change. Each square represents a differentially expressed transcript.

Furthermore, to get the overview of the pathways where the DETs are taking part in, the 1087 unigenes described above were further analyzed by MapMan software. The biotic stress and metabolism pathway visualization are shown in Figure 5. In the biotic stress visualization (Figure 5A), most of the genes whose expressions were altered by MES treatment were involved in signaling, proteolysis, and cell wall. Detail information of these pathways revealed that 23 of the genes in the signaling pathway belonged to the protein kinase signaling, and 21 belonged to calcium signaling. However, most of these genes in signaling were differentially expressed in An-LBs, except for eight genes altered in other tissues (Additional file 5). In proteolysis pathway, 32 of 56 protein degradation related genes were coding for the 26S proteasome complex, which mediates ubiquitin-dependent protein degradation. Interestingly, eight of the 56 protein degradation related genes were up-regulated in An-MBs, while most of the others were down-regulated in An-LBs (Additional file 5). This indicated that MES treatment might invoke activation of protein degradation process in the An-MBs of the MES-treated plants. In addition, a large number of cell wall related genes were down-regulated, this might be related to metabolism regulation instead of stress response, and it will be further analyzed below. In metabolism pathway visualization (Figure 5B), though the differentially expressed genes were dispersed in various primary and secondary metabolism pathways, a large number of genes were down-regulated in major and minor CHO, cell wall, and lipid metabolism in all four tissues. This result was consistent with the functional category and enrichment analysis mentioned above. Detail information of these pathways revealed that a large part of genes were differentially expressed in Ls, SBs, and An-MBs (Additional file 5). Interestingly, the few genes involved in amino acid metabolism were mainly up-regulated in An-LBs (Figure 5B, Additional file 5). Overall, these findings suggested that MES induced a tightly regulated gene network for metabolism reprogramming in the MES-treated plant anthers, especially for carbohydrate, cell wall, and lipid metabolism pathways.

### Expression changes of genes involved in carbohydrate, cell wall, and lipid metabolism

Combining functional category analysis results with cytological observation that severe damage occurred at late stage anthers (Figure 1J-N, R-T), we considered that most of the differentially expressed genes in An-LBs were the consequence of male sterility. On the contrary, early responses in SBs and An-MBs might provide some important clues to how low concentration of MES is inducing male sterility. Therefore, we paid more attention to the differentially expressed genes in Ls, SBs, and An-MBs. To further corroborate the view that expressions of carbohydrate, cell wall, and lipid metabolism-related genes were significantly altered by MES treatment during anther development process, detailed alternations of gene expressions were further analyzed in Ls, SBs, and An-MBs.

In the leaves of the MES-treated plants, the expressions of several carbohydrate metabolism-related genes, two cell wall-related genes, and one lipid metabolism-related gene were altered, of which three genes were localized in plastid (Table 2, Additional file 6). Four genes involved in starch biosynthesis and degradation pathway, including ADP glucose pyrophosphorylase (*AGP, AT5G48300*), phosphoglucomutase (*PGM, AT5G51820*), disproportionating enzyme (*DPE1,* 4-α-gluca-notransferase, *AT5G64860*), and alpha-glucan phosphorylase 2 (*PHS2, AT3G46970*), were down-regulated by approximately 2 ~ 3-fold. *AGP* has been shown to be one of the key regulatory enzymes catalyzing the first committed step of starch biosynthesis in higher plants [42,43]. *PGM* plays a pivotal role in the allocation of carbon between polysaccharide formation and energy production, and it is located in plastid [44]. *DPE1* and *PHS2* are enzymes catalyzing the breakdown of starch into maltose and glucose in the chloroplast at night [45,46]. In addition, another carbohydrate metabolism-related gene (*AT3G60750*), encoding transketolase in Calvin cycle, was down-regulated by 3.6-fold (Table 2). Importantly, a vital gene encoding sweet protein 11 (*AT3G48740*) was down-regulated by approximately 3-fold, which has been recently identified to mediate sucrose efflux in leaves as a key step for phloem transport [47]. These results indicated that the genes related to both starch biosynthesis and degradation processes as well as sugar transport were affected by MES treatment in leaves, suggesting that the transitory starch mobilization regulation network might be disturbed in the leaves of the MES-treated plants.

**Table 2:** **Representative differentially expressed genes involved in carbohydrate and lipid metabolism in Ls, SBs, and An-MBs**

**ProbeName**	**AGI**	**Symble**	**Short description**	Location*	**Fold change (MES/mock)**		
					**Ls**	**SBs**	**An-MBs**
**C-compound and carbohydrate metabolism**							
A_46_P058226	AT5G51820	PGM	Phosphoglucomutase	plastid	−3.1	-	-
A_46_P255759	AT5G64860	DPE1	Disproportionating enzyme	plastid	−2.6	-	-
A_46_P205459	AT5G48300	AGP	ADP glucose pyrophosphorylase 1		−2.6	-	-
A_46_P028531	AT3G46970	PHS2	Alpha-glucan phosphorylase 2		−2.2	-	-
A_46_P178359	AT3G60750		Transketolase	plastid	−3.6	-	-
A_46_P005266	AT2G05790		O-Glycosyl hydrolases family 17 protein		−3.1	-	-
A_46_P231464	AT3G48740		Sweet protein 11		−3.3	-	-
A_46_P140554	AT4G39800	MIPS1	Myo-inositol-1-phosphate synthase 1		-	−4.4	-
A_46_P352875	AT5G08380	AGAL1	Alpha-galactosidase 1		-	2.8	-
A_46_P050626	AT3G62410	CP12-2	CP12 domain-containing protein 2	plastid	-	-	7.9
**Cell wall**							
A_46_P147824	AT5G49360	BXL1	Beta-xylosidase 1		2.7	-	-
A_46_P289908	AT3G19450	CAD4	GroES-like zinc-binding alcohol dehydrogenase family protein		−2.4	-	-
A_46_P259534	AT5G49360	BXL1	Beta-xylosidase 1		-	−39.0	-
A_46_P165304	AT5G17200		Pectin lyase-like superfamily protein		-	−602.8	-
A_46_P132309	AT3G62170	VGDH2	VANGUARD 1 homolog 2	cell wall	-	-	−10273.7
A_46_P224439	AT3G07840		Pectin lyase-like superfamily protein		-	-	−6800.5
A_46_P132729	AT1G69940	PPME1	Pectin lyase-like superfamily protein	cell wall	-	-	−5579.7
A_46_P141564	AT5G48140		Pectin lyase-like superfamily protein		-	-	−4648.8
A_46_P058846	AT4G31370	FLA5	FASCICLIN-like arabinogalactan protein 5 precursor		-	-	−2337.3
A_46_P074236	AT2G47030	VGDH1	Plant invertase/pectin methylesterase inhibitor	cell wall	-	-	−1832.3
A_46_P302240	AT2G47040	VGD1	Plant invertase/pectin methylesterase inhibitor	cell wall	-	-	−319.9
A_46_P133334	AT1G02790	PGA4	Polygalacturonase 4		-	-	−30.0
A_46_P371475	AT3G07830		Pectin lyase-like superfamily protein		-	-	−22.66
A_46_P172054	AT1G63180	UGE3	UDP-D-glucose/UDP-D-galactose 4-epimerase 3		-	-	−19.5
A_46_P154974	AT4G30270	XTH24	Xyloglucan endotransglucosylase/hydrolase 24	cell wall	-	-	24.1
**Lipid, fatty acid and isoprenoid metabolism**							
A_46_P291948	AT1G45201	TLL1	Triacylglycerol lipase-like 1		−3.5	-	-
A_46_P011846	AT5G48880	KAT5	Peroxisomal 3-keto-acyl-CoA thiolase 2	mitochondrion	-	−5.9	-
A_46_P149169	AT3G61200		Thioesterase superfamily protein		-	−2.4	-
A_46_P162474	AT3G18280		Bifunctional inhibitor/lipid-transfer protein		-	2.0	-
A_46_P003226	AT1G20130		GDSL-like Lipase/Acylhydrolase superfamily protein	plastid	-	2.8	-
A_46_P239719	AT3G02040	SRG3	Senescence-related gene 3	plastid	-	9.7	-
A_46_P026421	AT2G25890		Oleosin family protein		-	-	−642.5
A_46_P109549	AT3G23510		Cyclopropane-fatty-acyl-phospholipid synthase		-	-	−9.9
A_46_P311390	AT4G35790	PLDDELTA	Phospholipase D delta		-	-	2.2
A_46_P095591	AT3G50660	CYP90B1	Cytochrome P450 superfamily protein		-	-	2.3
A_46_P357630	AT3G27660	OLEO4	Oleosin 4		-	-	4.6
A_46_P182574	AT1G30040	GA2OX2	Gibberellin 2-oxidase		-	-	4.7

*the localization information was from MIPS (Munich Information Center for Protein Sequences) website. Fold change was calculated between MES-treated plants and mock-treated plants; Ls, SBs, and An-MBs represents leaves, small buds, and anthers of middle buds, respectively; “-” represents that the gene was not significantly and differentially expressed in the specific tissue between the MES-treated and mock-treated plants. All the carbohydrate, cell wall, and lipid metabolism, and cellular transport related genes identified in this study were deposited in Additional file 6.

In SBs of the MES-treated plants, expression of four genes related to carbohydrate and cell wall metabolism, and several lipid related genes was altered, with one gene being localized in mitochondrion and two in plastid (Table 2, Additional file 6). Two genes related to cell wall metabolism, *BXL1* (beta-xylosidase, *AT5G49360*) and pectin lyase-like superfamily protein (*AT5G17200*) were down-regulated by 38.9-fold and 602.82-fold, respectively (Table 2). The latter gene exhibits polygalacturonase (PG) activity in cell wall metabolism and is highly expressed at early stages of flower development, which is essential for anther development [36]. In addition, expression of several lipid metabolism-related genes was altered, two of these genes being down-regulated and three up-regulated (Table 2). *KAT5 (AT5G48880)*, a thiolase, was down-regulated by 5.9-fold (Table 2). This gene is strongly expressed during flower development in *Arabidopsis* and can partially complements *KAT2,* which mutant exhibited partly male sterility [48]. In addition, one lipid transport gene (*AT3G18280*) and two lipid degradation genes (*AT1G20130*, *AT3G02040*) were up-regulated (Table 2).

In the An-MBs of the MES-treated plants, a cluster of cell wall-related genes, one gene involved in Calvin cycle, and several lipid related genes were differentially expressed (Table 2, Additional file 6). Most of the cell wall-related genes were significantly down-regulated, including cell wall precursor synthesis genes *FLA5* and *UGE3*, and pectin metabolism genes, such as polygalacturonase 4, the pectate lyase family protein, plant invertase/pectin methylesterase inhibitor superfamily, and VANGUARD 1. Previous studies revealed that pectin metabolism-related genes played important roles during late stages of pollen development [49,50]. Besides, another cell wall related-gene, UGE3 (*AT1G63180*) was down-regulated by 19.5-fold (Table 2). It is reported that *UGE2* together with *UGE3* affected pollen development [51]. In addition, several lipid metabolism-related genes were altered in the An-MBs of the MES-treated plants, two (*AT2G25890*, *AT2G25890*) of them involved in lipid biosynthesis were down regulated, and three (*AT4G35790*, *AT3G50660*, and *AT1G30040*) involved in lipid degradation were up regulated (Table 2).

The alteration of the expression profiles of the genes described above indicated that there was a dramatically transcriptome reprogramming on carbohydrate and lipid metabolism, especially on biosynthesis and degradation of cell wall and lipid in the anthers of the MES-treated rapeseed plants during anther development process. And the carbohydrate mobilization pathway in leaves was slightly repressed.

Functional categories enrichment analysis revealed that cellular transport, particularly material transport and electron transport function, was enriched in leaves and developing anthers under MES treatment (Figure 4C). It was found that detoxification-related genes such as ABC transporter, heavy metal transport, and MATE efflux family were up-regulated in the four tissues. Besides, metabolite transporters for sugars, peptides, amino acids, and nitrate were up-regulated in SBs and An-MBs (Additional file 5). However, energy metabolism-related genes about H^+^-exporting ATPase such as H(+)-ATPase 3 and H(+)-ATPase 9 were significantly down-regulated in the MES-treated plant anthers at late stage (Additional file 5). These results indicated that substrate transport pathway was activated, but energy production system might be repressed in the MES-treated plant anthers.

### Analysis of carbohydrate contents confirms that MES treatment influences carbon metabolism

**Figure 6:**
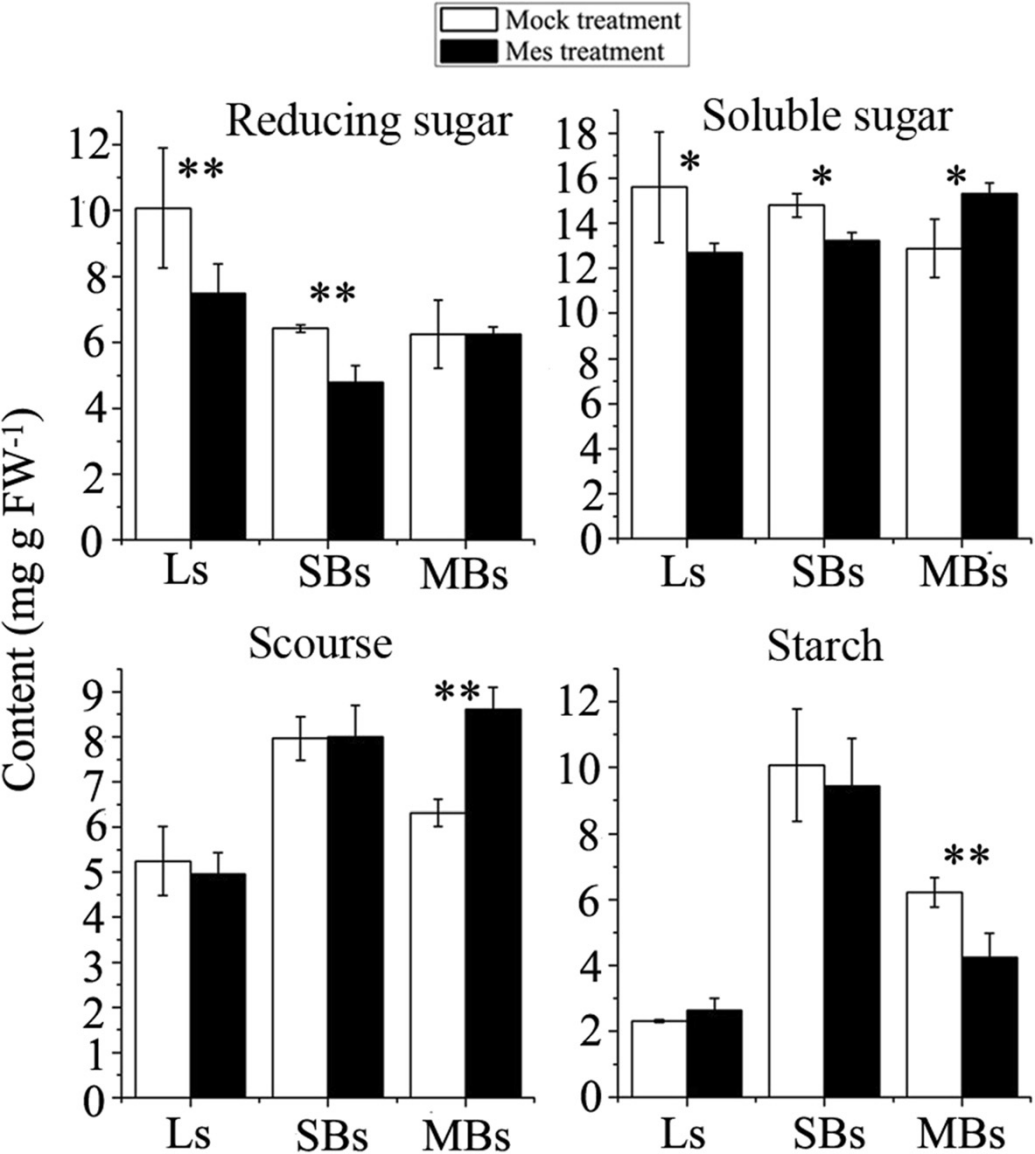
**Comparison of carbohydrate content between mock-treated and MES-treated plants.** Ls, young leaves from the main inflorescences; SBs, small buds with length less than 1 mm; MBs, middle buds with length of 1–3 mm in. *, **, represents significant difference at 0.05 level and at 0.01 level, respectively.

The ultrastructure and transcriptome analysis suggested that carbohydrate metabolism might be affected in the MES-treated plants. To confirm these findings, the carbohydrate contents, including soluble sugars, reducing sugars, sucrose, and starch, were analyzed in Ls, SBs, and MBs of both MES-treated and mock-treated plants, respectively (Figure 6). Compared with the mock-treated plants, the contents of soluble sugars and reducing sugars were significantly reduced in the Ls and SBs of the MES-treated plants. However, in the MBs of the MES-treated plants, the contents of soluble sugars and sucrose were increased but the starch content was decreased, compared with those in the same tissue of the mock-treated plants. These data confirmed that the carbohydrate metabolism in rapeseed leaves and anthers was significantly influenced by MES treatment, particularly in the late stage flower buds (MBs).

## Discussion

In this study, cytological observation, comparative transcriptome, and physiological analysis were conducted to reveal the mechanism of CHA-MES inducing male sterility in rapeseed. Cytological results showed that the ultrastructure of plastids/chloroplastids in the MES-treated plants was abnormal, and substances in plastids were deficient in pollen mother cells and tapetal cells but accumulated in epidermis and endothecium cells during anther development process. To gain a deeper insight into the effects of MES treatment on these processes, a comparative transcriptome analysis was performed between male sterility and fertility plant leaves and anthers. Functional analysis of the differentially expressed genes revealed that the carbohydrate, cell wall, lipid metabolism, and cellular transport processes were enriched. Detailed expression of these genes was analyzed also in leaves, small buds, and anthers from middle buds. Carbohydrate content analysis further confirmed the results of cytological observation and transcriptome analysis.

### MES treatment disturbes plastid and mitochondrion functions, probably through acetolactate synthetase (ALS)

MES is an inhibitor of ALS [9], localized in the plastid/chloroplast [10]. ALS is universally expressed in plant tissues, including leaves, seeds, young siliques, and flower buds, but its highest expression level is in mature pollen grains in *Arabidopsis* (Additional file 7A, B). Co-expression analysis of *Arabidopsis ALS* (*At3g48560*) revealed a network of 10 genes directly or indirectly related to *ALS* (Additional file 7C): plastidic pyruvate kinase beta subunit 1 (*At5g52920*), acetyl Co-enzyme a carboxylase biotin carboxylase subunit (*At5g35360*), acetyl Co-enzyme a carboxylase carboxyltransferase alpha subunit (*At2g38040*), ketol-acid reductoisomerase (*At3g58610*), pyruvate dehydrogenase E1 alpha (*At1g01090*), semialdehyde dehydrogenase family protein (*At1g14810*), isopropylmalate dehydrogenase 2 (*At1g80560*), adenylosuccinate synthase (*At3g57610*), transketolase family protein (*At2g34590*), and pyruvate kinase family protein (*At3g22960*). Most of these genes are expressed in the plastid (six genes) or mitochondrion (three genes) and are involved in five main pathways, including biosynthesis of secondary metabolites; purine metabolism; valine, leucine, and isoleucine biosynthesis, and two carbohydrate-related pathways (pyruvate metabolism and glycolysis/gluconeogenesis). In flowering plants, plastids are the primary organelles that accumulate carbohydrates and lipid compounds in the tapetum [52] and perform essential metabolic functions in the synthesis of lipid and secondary products [53]. In the present study, the MES-treated plants showed an abnormal plastid development from the PMC stage, and the plastid/chloroplastid structure exhibited obvious defects in tapetal cells, epidermis, and endothecium cells at the vacuolated-microspore stage (Figure 1). In addition, subcellular localization analysis of the differentially expressed genes showed that most of them were localized in the plastid and mitochondrion (Figure 4A). We proposed that MES treatment might disturb the normal functions of the plastid and mitochondrion, two functionally communicating organelles in plant cells, through targeting *ALS*.

### MES treatment influences carbohydrate and lipid metabolism during anther development

In this study, TEM analysis showed substrate deficient to form normal plastids containing starch or lipids in male gametophyte cells, and an abnormal accumulation of large starch granules in epidermis cells in the MES-treated anthers at the vacuolated-microspore stage (Figure 1). These results suggested that there was a metabolism block during anther development process in the MES-treated plants. Furthermore, functional categories and pathway analysis of differentially expressed genes related to anther development showed that carbohydrate, cell wall, and lipid metabolism pathways were significantly affected in the MES-treated plants (Figure 4C, Figure 5, and Additional file 5). Detailed analysis of gene expression alternation showed that starch and sucrose metabolism-related genes (*AGP, PGM, DPE1,* and *PHS2*) were down-regulated in young leaves of the main inflorescences in the MES-treated plants. In addition, an important gene (*sweet protein 11, AT3G48740*) encoding a protein for sucrose phloem transport was also down-regulated (Table 2, Additional file 5). In contrast, several substrate transport-related genes (including sugar, lipid, peptide, amino acid, and nitrate transports) were up-regulated in the early development stage anthers (SBs) of the MES-treated plants. Furthermore, lipid biosynthesis related genes were down-regulated during anther development process, companying with lipid degradation related genes up-regulated (Additional file 5). This was consistent with our cytological analysis that two lipid-storage plastids, elaioplast and tapetosome, were impaired at different degrees under MES treatment (Figure 1G-Q). Therefore, carbohydrate transport from vegetative to reproductive tissues was likely to be slightly suppressed at early anther developmental stages, and carbohydrate and lipid metabolism was abnormal in the MES-treated plants anthers.

Pollen wall consisting of intine and exine needs sugars and lipids for its formation. Intine is secreted by the microspore when released from the callose wall, comprising cellulose, pectin, and various proteins [54]. Previous investigations indicated that at least three types of cell wall-related enzymes functioned in pollen development process, including beta-1,3-glucanase [32,33], endocellulase [34,35], and polygalacturonase (PG) [36]. Several genes responsible for genic male sterility in *B. napus* mutants were map-based cloned, and were found to be lipid metabolism related genes [55-57]. It was reported that the *PG* family protein was associated with pollen intine development in *B. campestris*, and its mutant displayed male sterility [49,50]. Recently, analysis of gene expression profiles between genic male sterile plants and their fertile counterparts in *B. napus* [58] and cotton [59] by high-throughput digital gene expression technique revealed that numerous genes involved in starch and sucrose metabolism were also altered. These suggested that expression alteration of the genes in carbohydrate and lipid metabolism could result in male sterility in plants. In this study, several pectin-related genes were down-regulated in the developing anther of the MES-treated plants. Furthermore, lipid-transfer protein and cyclopropane-fatty-acyl-phospholipid synthase (*CAF, AT3G23510*), associated with cell wall and membrane biogenesis, were down-regulated. So both carbohydrate and lipid nutrient were deficient in the developing anthers of MES-treated plants, which might contribute to rapeseed male sterility.

Furthermore, in this study, *H(+)-ATPase 3* and *H(+)-ATPase 9* were down-regulated in Ls and An-MBs (Table 2) from the MES-treated plants, and several other ATPase-related genes were down regulated in An-LBs (Additional file 5). We inferred that down regulation of these ATPases might be one of the consequent effects caused by MES treatment, which could also contribute to rapeseed male sterility, because mitochondrial gene rearrangements affecting ATP production have been reported to be the reason for cytoplasmic male sterility (CMS) [60].

Taken together, carbohydrate and lipid metabolism was blocked in the MES-treated rapeseed plants, which may be the effect of MES treatment mainly responsible for *B. napus* male sterility.

### Similar action mode of CHA-MES as that of ALS inhibitor herbicides but different organ effects

Till date, several ALS inhibitor herbicides, including tribenuron-methyl, amidosulphuron, and monosulfuron-ester sodium, have been found to induce complete male sterility in rapeseed when applied at a concentration below 1% of that required for their herbicidal activities [6,8,11]. To investigate whether these ALS inhibitor herbicides work as CHA in the same manner as herbicides, we compared our results with previous reports on the action mode of herbicides. When ALS inhibitors are applied as herbicides, following the inhibition of ALS, plants respond quickly to renew BCAAs level by increasing protein turnover, so that the BCAA pool does not decline to a level that would affect protein synthesis, leading to an increase in the total free amino acid pool [15-18,61]. This phenomenon was also observed in the ALS inhibitor inducing male sterility plants [62]. In addition, a rapid increase in the level of carbohydrate in leaves of plants treated with ALS inhibitors was reported [19], and this effect was related to a decreased photoassimilate translocation to sink tissues [20] due to a decreased sink strength [21]. In this study, we detected a decrease in the content of soluble and reducing sugars and a slight but not significant increase in starch content in the leaves of the MES-treated plants. While in flowering organs of the MES-treated plants, the soluble sugars content was decreased at early stage (SBs) but increased at late stage (MBs), starch content in both SBs and MBs was continuously decreased. These results suggested that carbohydrates translocation between vegetative and reproductive organs was slightly blocked in the MES-treated plants, but this seemed not to be the essential reason for inducing male sterility. Because late increase of soluble sugars content in MBs and continuous decrease in starch content from SBs to MBs indicated that the sugars did transport to reproductive organs from leaves, but the assimilation of carbon in flowers was weak, which might be contributed to male sterility.

Qian *et al.*(2011) [63] reported that imazethapyr (IM) affected carbohydrate metabolism in chloroplasts, including starch and other sugars, and IM treatment resulted in the accumulation of glucose, maltose, and sucrose in the cytoplasm or chloroplast and disturbed carbohydrate utilization. They confirmed that metabolic pathways, including amino acid metabolism, photosynthesis, starch and sugar metabolism, and the tricarboxylic acid cycle were altered in IM-treated rice [64]. Manabe *et al.*(2007) [24] confirmed that CSR1, the catalytic subunit of ALS, was the sole target of imidazolinone herbicide in *A. thaliana* using microarray analysis*.* Das *et al.* (2010) [23] identified 478 genes significantly and coordinately regulated by four ALS-inhibiting herbicides, including one imidazolinone, one triazolopyrimidine, and two sulfonylureas at the EC_50_ concentration. Among their 478 genes identified, only 28 with the same AGI number were differentially expressed in our data, functionally involved in photosynthesis, OPP (oxidative pentose phosphate) cycle, TCA, mitochondrial electron transport/ATP synthesis, secondary metabolism and stress and so on. However, the function categories influenced by ALS inhibitors used as herbicides (Das’s study) and CHA (our study) were similar, though the specific genes affected were different. We assumed that this was caused by two main reasons. First is that different plant species and different tissues (organs) were tested in both studies. In our study, we used rapeseed flower buds, anthers, and leaves from the main inflorescences, including vegetative and reproductive tissues, whereas, in Das’s study, they used leaves of 14-day-old seedlings of *A. thaliana* as experimental materials. Second is the very different concentration of ALS inhibitor used in the two studies. We treated rapeseed plants with 0.24 g ha^−1^ MES (approximately 1% of MES concentration required to control broadleaf weeds in wheat field) for inducing male sterility. However, Das *et al.* (2010) [23] treated *A. thaliana* plants with four ALS-inhibiting herbicides at their EC_50_ concentrations, ranging from 0.131 g ha^−1^ for sulfometuron-methyl to 0.586 g ha^−1^ for primisulfuron-methyl.

Taken together, the general action mode of ALS inhibitor herbicides on plants was seemly the same whether they were used as herbicides or as CHAs at very low concentration. However, why vegetative and reproductive organs exhibited different responses (vegetative normal but reproductive male sterility) to the ALS inhibitor MES using as CHA at low concentration? Previous study indicated that ALS expression level is the highest in mature pollen grains in *Arabidopsis* (Additional file 7), so the developing anthers might be more sensitive to MES treatment than other vegetative tissues (organs). We speculated that two aspect effects might contribute to this phenomenon. Firstly, the ALS inhibition might immediately affect synthesis of some anther specific BCAAs-enriched enzymes or proteins, or facilitate the degradation of BCAAs-enriched enzymes or proteins to renew BACCs levels, considering that the free amino acids content was higher in ALS inhibitor inducing male sterility plant anthers than in the control plant anthers [62], and several genes involved in protein degradation were up-regulated in An-MBs in this study (Additional file 5). Secondly, when ALS was inhibited in developing anthers, large amount of the substrate (pyruvate and 2-ketobutyric acid) might be accumulated and then might disturb other metabolism pathways such as carbohydrate and lipid metabolism through metabolism reprogramming, which was essential for anther development. Since carbon skeletons are necessary to synthesis several fundamental materials including amino acids, fatty acids, and secondary metabolites. Thus, low concentration of MES induces specifically male sterility, but has no serious effect on vegetative tissues (organs). However, all these inferences need further experiments to verify.

### Putative action of MES-induced male sterility in *B. napus*

**Figure 7:**
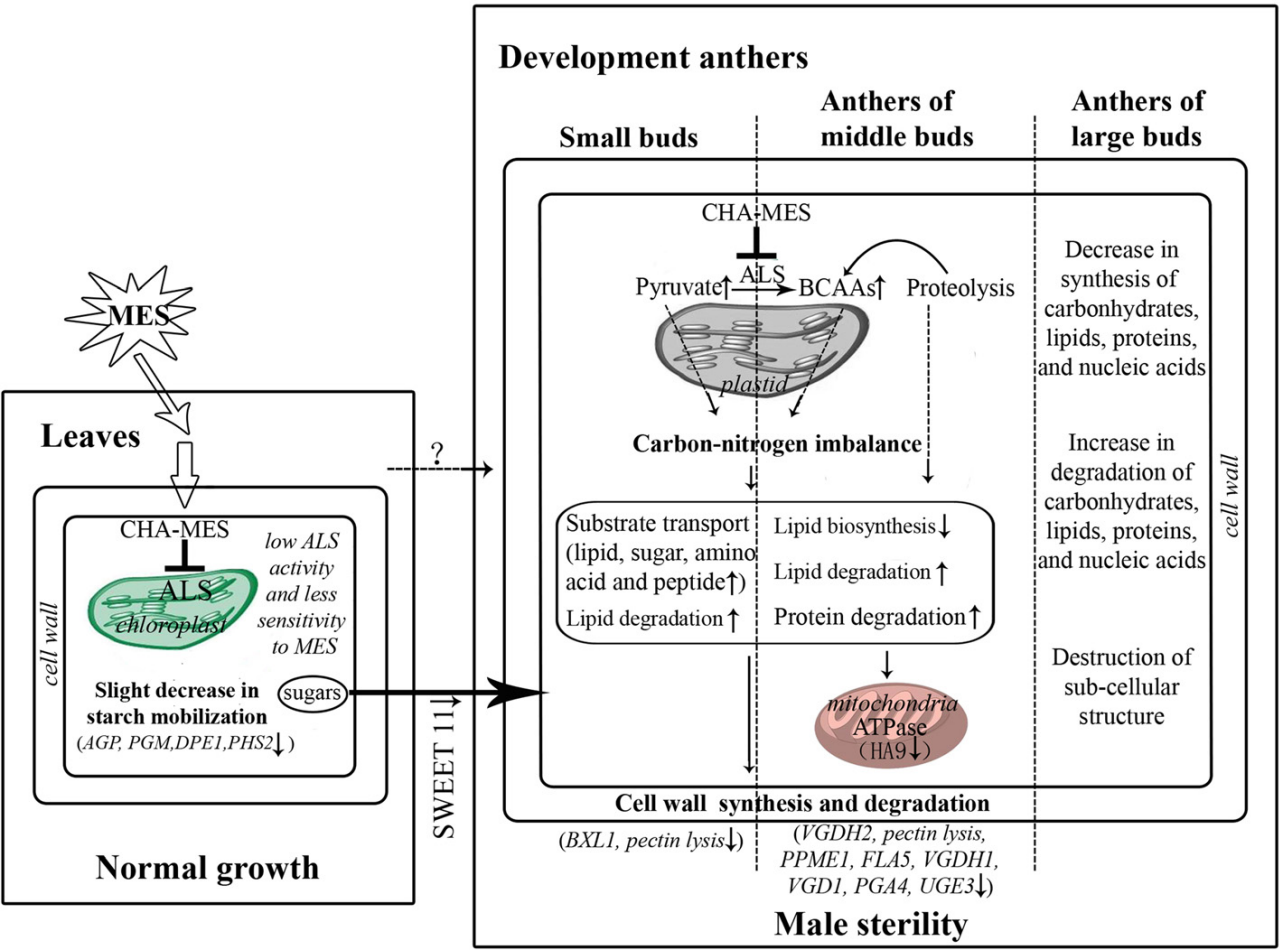
**A putative action model for MES-treatment inducing male sterility.** Some important functions and genes affected by MES treatment in leaf tissue (left rectangle) and developing anther tissues (right rectangle) are listed (see text for details). Two vertical dashed lines in the right rectangle separate three anther tissues. ‘↑’ after function categories or genes means up-regulation; ‘↓’ after function categories or genes means down-regulation. Two aspects of putative reasons for carbohydrate and lipid metabolism alteration were showed by dashed arrows ‘?’ represents unclear MES transport pathway. MES, Monosulfuron Ester Sodium; ALS, acetolactate synthase; BCAAs: Branch-Chain Amino Acids; AGP, ADP glucose pyrophosphorylase; PMG, Phosphoglucomutase; DPE1, Disproportionating enzyme; PHS2, alpha-glucan phosphorylase 2; SWEET 11, Nodulin MtN3 family protein; BXL1, beta-xylosidase; pectin lyases, pectin lyase superfamily protein; VGDH2, VANGUARD 1 homolog 2; PPME1: Pectin lyase-like superfamily protein; FLA5: FASCICLIN-like arabinogalactan protein 5 precursor; VGDH1: Plant invertase/pectin methylesterase inhibitor; VGD1: Plant invertase/pectin methylesterase inhibitor; PGA4, Polygalacturonase 4; UGE3, UDP-D-glucose/UDP-D-galactose 4-epimerase 3; HA9, H(+)-ATPase 9.

Combining with ultrastructural cytological observation, systematic comparative transcriptome analysis,and metabolic analysis, we updated the simple action model of CHA-MES inducing male sterility in rapeseed, which was proposed in our previous investigation [8] (Figure 7). In this study, we speculated that, following the inhibition of ALS, two aspect of effects might be caused in development anthers by MES treatment at low concentration: One was proteolysis of some essential enzymes related with carbohydrate and lipid metabolism to renew BCAAs level; another was accumulation of pyruvate, this disturbing the normal level of carbon skeleton substrates in plastids/chloroplasts and leading to pyruvate diverting to the closely related metabolism pathways such as carbohydrate and lipid metabolism. Therefore, the metabolism of carbohydrate and lipids, two types of macromolecules playing crucial roles during anther development,was blocked during anther development process in the MES-treated plants. Furthermore, mitochondria were functionally disturbed by MES treatment at low concentration based on our comparative transcriptome analysis. This organelle was functionally coupled with the plastid/chloroplast and supplies with metabolism substrates and energy for plant development. Taken together, carbohydrate and lipid metabolism blocks in the development anthers of the MES-treated rapeseed plants might be mainly responsible for *B. napus* male sterility induced by MES treatment, along with energy deficiency and perturbed network regulation. In leaves, though several genes involved in starch and sucrose metabolism were detected to be down-regulated in the MES-treated plants, and the content of soluble sugars decreased and starch content slightly increased, vegetative tissues of the MES-treated plants exhibited no obvious difference to those of the mock-treated plants. However, how the inhibition of ALS in anthers actually affected carbohydrate and lipid metabolism in development anthers remains to be further study.

## Conclusions

This study carried out a systematic analysis of effects caused by CHA-MES treatment at ultrastructure, transcriptome, and physiological levels, which revealed that the carbohydrate and lipid metabolism was altered in rapeseed male sterility plants induced by MES treatment at low concentration. Accordingly, we proposed a simple action model for CHA-MES inducing male sterility in *B. napus*. These results will provide some clues to the mechanism of MES inducing male sterility, and give insights into the complex gene regulation network during anther development. Besides, these results might provide more potential targets for developing new male sterility inducing CHAs and for genetic manipulation in rapeseed breeding.

## Methods

### Plant material and experimental setup for MES treatment

The rapeseed cultivar ‘Zhongshuang No.9’, developed by the Oil Crops Research Institute of Chinese Academy of Agricultural Sciences (Wuhan, China) and selfed for eight generations before being used in the present experiment, was planted in the experimental field of Northwest A&F University, Yangling, Shaanxi, China (longitude 108°E, latitude 34°15′N) during a natural growth season on 23rd September 2009. Optimal agronomic practices were followed.

The experimental plot contained approximately 2,400 plants grown in 120 rows (2-m long each), with a space of 50 cm between rows and 10 cm between plants within a row. When the rapeseed plants were at the bolting stage with the longest floral bud being ≤2 mm, the plot was divided into two groups: MES-treated group and mock-treated group, each containing 60 rows. MES was kindly provided by Professor Zhengming Li of NanKai University, Tianjin, China. The plants of MES-treated group were foliar sprayed with 0.1 μg mL^−1^ MES solution containing 50 ppm DMF and 5 ppm Tween 80 for approximately 15 mL per plant (approximately 1% of the concentration that is required for its herbicide action in wheat field to control broadleaf weeds) for inducing male sterility during the entire flowering period without affecting the growth and development of other rapeseed plants tissues. Meanwhile, the plants of the mock-treated group were foliar sprayed with the same amount of solution only containing 50 ppm DMF and 5 ppm Tween 80 as the control.

### Cytological study

The protocol used for cytological studies was described in the previous report [8]. In brief, when the fertility of the first opened flower of each MES-treated plant was visually detectable for male sterility, the main inflorescences of uniform plants in the MES-treated and mock-treated groups were collected into plastic bags and quickly transported to the laboratory on ice. Acetocarmine staining was performed to examine the correlation of the microspore developmental stage with the bud length. Bud samples of the MES-treated and mock-treated plants at different microspore developmental stages were treated according to González-Melendi *et al.* (2008) [65] for cytological observation. After treatment, the specimens were sectioned with Ultramicrotome Leica EM UC7 (Leica Microsystems, Germany). Ultrathin sections (70 nm) were observed and photographed with a transmission electron microscope (JEM-1230, JEOl, Tokyo, Japan) on 600 mesh formvar-coated copper grids.

### Plant sample collection for microarray study

Plant sample collection for microarray study was the same as that for the previous proteomic study [8]. In brief, based on cytological observation results of acetocarmine staining, the collected inflorescence samples of the MES-treated and mock-treated groups were classified into three subgroups according to their bud length, namely small buds (SBs) with length below 1 mm (before and during the pollen mother cell (PMC) stage), medium buds (MBs) with 1–3 mm in length (from meiosis to the early-uninucleate-microspore stage), and large buds (LBs) with length over 3 mm (from the vacuolated-microspore to the mature-pollen stages). In the MB and LB subgroups, anthers were dissected from the buds, designed as An-MBs and An-LBs, respectively. Young leaves (Ls) from the main inflorescences of the MES-treated or mock-treated plants were also collected as vegetative tissue control. All samples were prepared on ice, immediately frozen in liquid nitrogen and then stored at −80°C for later use. Mixture samples collected from every 20 rows of the MES-treated or mock-treated plants were used as one biological replicate, and three independent biological replicates were then prepared for each sample.

### Microarray experiment and data acquisition

The Agilent Single Channel *Brassica* Oligo Microarray (4 × 44 K) was used in this study; the chip contains 43,803 probe sets designed on the basis of ESTs of *B. napus*, mRNAs, and predicted gene sequences from databases such as NCBI, TIGRI, and UniGene. Total RNAs of 24 samples, four pair tissues from the MES-treated and mock-treated plants with three biological replicates, were extracted using TRIzol reagent (Invitrogen Life Technologies, Carlsbad, CA, US) and purified using the QIAGEN RNeasy® Mini Kit (QIAGEN, GmBH, Germany). In total, 1.65 μg cRNA was used for hybridization, and washing, staining, and scanning were performed according to instructions. Three independent biological replicates were included in each microarray experiment. The hybridization signals were normalised by Quantile algorithm [66] using Gene Spring Software 11.0 (Agilent technologies, Santa Clara, CA, US) and log2 transformed.

For the identification of DETs involved in microgametogenesis between the MES-treated and mock-treated groups, two sets of Student’s t-test comparisons were performed (Figure 3). First, comparisons within groups, named vertical comparisons, were performed. Pairwise comparisons of Student’s t-test between tissues (organs) were performed within the mock-treated group and MES-treated group to detect DETs related to anther development under mock treatment (control) (fertile) and MES treatment (male sterile) conditions (Figure 3A). Second, comparisons between groups, named horizontal comparisons, were performed. These set of comparisons were performed in the four pairs of corresponding tissues (organs) between the MES-treated and mock-treated groups to identify DETs related to MES treatment (Figure 3B). The results of all these comparisons were filtered with the constraint of fold change ≥ 2 and p-value ≤0.001. To focus on genes presumably related to anther development, which are influenced by MES treatment, the common DETs in two sets of comparisons (the red and green parts in Figure 3C) were considered to be anther development-related genes affected by MES treatment. Microarray data were deposited to the database of the National Center for Biotechnology Information (NCBI) with the accession number GSE53468 (http://www.ncbi.nlm.nih.gov/geo/query/acc.cgi??).

### Annotation and functional analysis

The identified differently expressed transcripts (DETs) of *B. napus* were annotated by BLASTN against TAIR (http://www.arabidopsis.org/Blast/index.jsp) in the present study. The unigenes (AGI identifers) with BLASTN expectation values (E-values) <10^−5^ [57] were used to annotate the target transcripts. Subsequently, the DETs with AGI identifers were used for further functional analysis.

To categorize differentially expressed genes based on their subcellular localization and biological functions, the rapeseed DETs with unique AGIs were submitted to the Munich Information Center for Protein Sequences (MIPS) catalogue of *A. thaliana* genome [67]. In addition, pathway visualization and analysis was performed using MapMan [68]. Furthermore, to obtain more cell wall biogenesis and lipid metabolism related genes, the differentially expressed genes with AGIs were also compared to Cell Wall Genomics database (http://cellwall.genomics.purdue.edu/families/index.html) and The Arabidopsis Lipid Gene Database (http://lipids.plantbiology.msu.edu/), respectively.

### Data validation by quantitative real-time PCR (qRT-PCR)

To confirm the differential expression pattern of DETs detected in the microarray experiments, qRT-PCR analyses were performed. Gene-specific primers were designed according to the reference unigene sequences (Additional file 8). Total RNAs were isolated using TRIzol reagent from the same plant samples as those used in the above mentioned microarray experiment. For each sample, cDNA was generated from 1 mg of total RNA using the MMLV Reverse Transcriptase TIANScript RT Kit (TIANGEN, China) according to instructions. The *B. napus β-actin* (accession no. AF111812.1) gene was used as a reference [69], and the relative gene expression levels were calculated using the2^−ΔΔCt^ method [70]. The results were obtained from three biologically independent experiments.

### Carbohydrate content analysis

To determine the composition changes in carbohydrate, such as soluble sugars, sucrose, and starch contents in both MES-treated and mock-treated groups, we analyzed three independent biological replicates for each tissue. The sugar content was measured according to Dorion *et al*. (1996) [71]. 1.0–3.0 g (fresh weight) of each tissue tested was used for sugar extraction and for starch content analysis. Total soluble sugars and reducing sugars were determined using the anthrone method [72] and 3,5-dinitrosalicylic acid method [73], respectively. The difference between the total soluble sugars content and the reducing sugars content was the amount of non-reducing sugars, which was recognized to be sucrose. The starch content was determined by hydrolyzing it to soluble sugars and calculated [71].

## Additional files

Additional file 1:
**Correlation coefficients between the three biological replicates of 24 samples.**
Additional file 2:
**Reproducibility of microarray experiments assessed by qRT-PCR.**
Additional file 3:
**List of 1501 differentially expressed transcripts (DETs) identified between the MES-treated and the mock-treated plants.**
Additional file 4:
**Annotation result of the 1501 differentially expressed transcripts (DETs) according to Arabidopsis Information Resource (TAIR).**
Additional file 5:
**Functional pathways of significantly differentially regulated genes assigned by MapMan.**
Additional file 6:
**Carbohydrate metabolism, cell wall formation, lipid metabolism, and cellular transport related genes influenced by MES treatment.**
Additional file 7:
**ALS subcellular localization, tissue expression profile, and co-expression analysis based on the information from TAIR.**
Additional file 8:
**Primers for quantitative real time PCR (qRT-PCR).**

## Abbreviations

ALS: acetolactate synthase
AHAS: acetohydroxyacid synthase
TAIR: *Arabidopsis* Information Resource
BRAD: *Brassica* Database
CHA: Chemical hybridization agent
DETs: differentially expressed transcripts
IM: imazethapyr
MES: monosulfuron ester sodium
MIPS: Munich Information Center for Protein Sequences
PMC: Pollen Mother Cell
