# Supplementary material for: Comparative transcriptome analysis reveals carbohydrate and lipid metabolism blocks in Brassica napus L. male sterility induced by the chemical hybridization agent monosulfuron ester sodium
Source: BMC Genomics. 2015 Mar 17;16(1):206. doi: 10.1186/s12864-015-1388-5 (PMC4376087; doi:10.1186/s12864-015-1388-5)
Supplement: Additional file 1: — Correlation coefficients between the three biological replicates of 24 samples. [file 12864_2015_1388_MOESM1_ESM.docx]

Additional file 1: Correlation coefficients between the three biological replicates of 24 samples

|  |  |  |  |  |  |
| --- | --- | --- | --- | --- | --- |
| Mo-Ls1 |  |  | Tr-Ls1 |  |  |
| Mo-Ls2 | 0.9906 |  | Tr-Ls2 | 0.9690 |  |
| Mo-Ls3 | 0.9802 | 0.9720 | Tr-Ls3 | 0.9118 | 0.8927 |
|  |  |  |  |  |  |
| Mo-SBs1 |  |  | Tr-SBs1 |  |  |
| Mo-SBs2 | 0.9704 |  | Tr-SBs2 | 0.9724 |  |
| Mo-SBs3 | 0.9635 | 0.9519 | Tr-SBs3 | 0.9813 | 0.9643 |
|  |  |  |  |  |  |
| Mo-An-MBs1 |  |  | Tr-An-MBs1 |  |  |
| Mo-An-MBs2 | 0.9704 |  | Tr-An-MBs2 | 0.8495 |  |
| Mo-An-MBs3 | 0.9635 | 0.9519 | Tr-An-MBs3 | 0.9469 | 0.9076 |
|  |  |  |  |  |  |
| Mo-An-LBs1 |  |  | Tr-An-LBs1 |  |  |
| Mo-An-LBs2 | 0.9657 |  | Tr-An-LBs2 | 0.9340 |  |
| Mo-An-LBs3 | 0.9866 | 0.9424 | Tr-An-LBs3 | 0.9428 | 0.9245 |

Mo-Ls and Tr-Ls: leaves from the main inflorescences in the mock-treated and MES-treated plants, respectively; Mo-SBs and Tr-SBs: the small buds less than 1mm in length containing microgametocyte before and during pollen mother stage in the mock-treated and MES-treated plants, respectively; Mo-An-MBs and Tr-An-MBs: the anthers from middle buds with length between 1mm and 3mm containing microgametocyte from meiosis to early uninucleate microspore stage in the mock-treated and MES-treated plants, respectively; Mo-An-LBs and Tr-An-LBs: anthers from large buds more than 3mm in length containing microgametocyte from voacuolated stage to mature pollen stage in the mock-treated and MES-treated plants, respectively.

1,2,3: represents three independent biological replicates.
