# Supplementary material for: Comparative transcriptome analysis reveals carbohydrate and lipid metabolism blocks in Brassica napus L. male sterility induced by the chemical hybridization agent monosulfuron ester sodium
Source: BMC Genomics. 2015 Mar 17;16(1):206. doi: 10.1186/s12864-015-1388-5 (PMC4376087; doi:10.1186/s12864-015-1388-5)
Supplement: Additional file 2: — Reproducibility of microarray experiments assessed by qRT-PCR. [file 12864_2015_1388_MOESM2_ESM.docx]

Additional file 2: Reproducibility of microarray experiment assessed by qRT-PCR
