# Supplementary material for: Comparative transcriptome analysis reveals carbohydrate and lipid metabolism blocks in Brassica napus L. male sterility induced by the chemical hybridization agent monosulfuron ester sodium
Source: BMC Genomics. 2015 Mar 17;16(1):206. doi: 10.1186/s12864-015-1388-5 (PMC4376087; doi:10.1186/s12864-015-1388-5)
Supplement: Additional file 4: — Annotation result of the 1501 differentially expressed transcripts (DETs) according to Arabidopsis Information Resource (TAIR). [file 12864_2015_1388_MOESM4_ESM.docx]

Additional file 4: Annotation result of the 1501 differentially expressed transcripts (DETs) according to *Arabidopsis* Information Resource (TAIR)

| Tissues/organs | No. of DETs | No. of AGI* | No. of unique AGI | Not annotated DETs |
| --- | --- | --- | --- | --- |
| Ls | 77 | 74 | 65 | 3 |
| SBs | 67 | 61 | 59 | 6 |
| An-MBs | 161 | 150 | 126 | 11 |
| An-LBs | 1238 | 1130 | 897 | 108 |
| total | 1501 | 1379 | 1087 | 122 |

*: AGI represents the Arabidopsis genes identifer obtained from TAIR.

The *B. napus* DETs were conducted BLASTN against TAIR (<http://www.arabidopsis.org/Blast/index.jsp>), and the *A. thaliana* genes (AGI) with high similarity (E-value < 10−5) were collected.
