# Supplementary material for: Comparative transcriptome analysis reveals carbohydrate and lipid metabolism blocks in Brassica napus L. male sterility induced by the chemical hybridization agent monosulfuron ester sodium
Source: BMC Genomics. 2015 Mar 17;16(1):206. doi: 10.1186/s12864-015-1388-5 (PMC4376087; doi:10.1186/s12864-015-1388-5)
Supplement: Additional file 7: — ALS subcellular localization, tissue expression profile, and co-expression analysis based on the information from TAIR. [file 12864_2015_1388_MOESM7_ESM.docx]

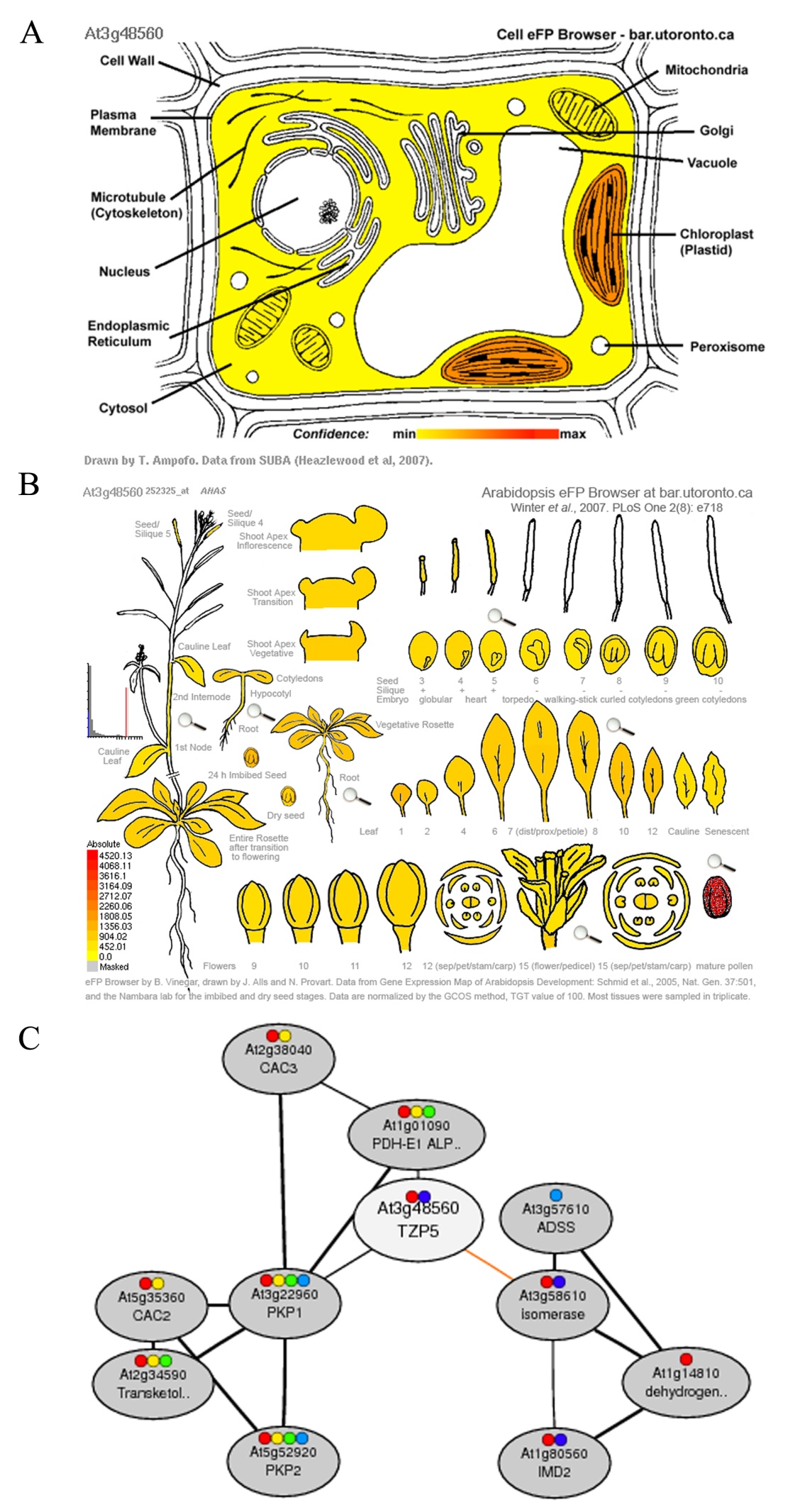


Additional file 7: ALS subcellular localization (A), tissue expression profile (B) and co-expression network analysis (C) based on the information from TAIR

ALS localized in chloroplast/plastid, and universally expressed in plant tissues, including leaves, seeds, young siliques, flower buds and showed the highest expression level in mature pollen grains in *Arabidopsis.*

At5g52920, plastidic pyruvate kinase beta subunit 1; At5g35360, acetyl Co-enzyme a carboxylase biotin carboxylase subunit; At2g38040, acetyl Co-enzyme a carboxylase carboxyltransferase alpha subunit; At3g58610, ketol-acid reductoisomerase; At1g01090, pyruvate dehydrogenase E1 alpha; At1g14810, semialdehyde dehydrogenase family protein; At1g80560, isopropylmalate dehydrogenase 2; At3g57610, adenylosuccinate synthase; At2g34590, Transketolase family protein; At3g22960, Pyruvate kinase family protein.

Color spots represent different pathways the co-expressed genes involved. Red, biosynthesis of secondary metabolites; yellow, pyruvate metabolism; green, glycolysis / gluconeogenesis; light blue, purine metabolism; dark blue, valine, leucine and isoleucine biosynthesis
